# Dependence of *Z* Parameter for Tensile Strength of Multi-Layered Interphase in Polymer Nanocomposites to Material and Interphase Properties

**DOI:** 10.1186/s11671-017-1830-5

**Published:** 2017-01-17

**Authors:** Yasser Zare, Kyong Yop Rhee

**Affiliations:** 1 Young Researchers and Elites Club, Science and Research Branch, Islamic Azad University, Tehran, Iran; 2 Department of Mechanical Engineering, College of Engineering, Kyung Hee University, Yongin, 446-701 Republic of Korea

**Keywords:** Polymer nanocomposites, Interphase layer, Material properties, Tensile strength

## Abstract

In this work, the *Z* interphase parameter which determines the tensile strength of interphase layers in polymer nanocomposites is presented as a function of various material and interphase properties. In this regard, the simple Pukanszky model for tensile strength of polymer nanocomposites is applied and the dependency of *Z* to different characteristics of constituents and interphase are illustrated by contour plots. The interphase strength (*σ*_i_) and *B* interfacial parameter in Pukanszky model show direct links with *Z* parameter. Also, it is found that the volume fractions of nanoparticles and interphase reveal dissimilar effects on *Z*. A high *Z* is obtained by a low nanoparticle volume fraction and high content of interphase, but the best values of *Z* are associated with the level of *B* parameter.

## Background

The exceptional improvements of mechanical properties at low nanofiller contents have introduced significant interest in the use of nanoparticles in polymer matrices [1–6]. The different types of nanofillers have been used to strengthen and toughen the polymers. The most interesting aspect related to nanofiller is that they can improve the properties of polymers at very low filler concentrations compared to micro-particles and fibers [7–9]. This phenomenon can be described as nanoeffect which is the interactions at the atomic scale. When the particle size decreases to the nanoscale, the specific surface area rapidly increases, making the surface properties as the dominant factors and providing unique characteristics with widespread applications in many industrial parts. Additionally, when the filler size is similar to that of polymer chains, molecular interactions between nanoparticles and polymer matrix produce a third phase as interphase which has different properties from both polymer and nanoparticles [10, 11]. The properties of interphase play a main role in the level of dissipated energy by different damaging mechanisms which take place at the nanoscale [12, 13]. As a result, the mechanical properties of the nanocomposites significantly depend on the interphase level.

Many researchers have tried to characterize the interphase properties by modeling of the general properties of nanocomposites because the interphase is affected by many factors, and it cannot be characterized by simple techniques [14, 15]. The theoretical surveys in the recent years provided a large amount of information about interphase and interfacial interactions in polymer nanocomposites.

Several researchers have considered a multi-layered interphase in polymer nanocomposites. They assumed that each layer in interphase has different properties from others. The characteristics of interphase layers were hypothetically studied and their influences on the nanocomposite behavior were discussed in many papers [16–18]. In one study, the thickness of interphase was assumed as a characteristic length scale and the main effects of interphase on stiffness and yield stress of polymer nanocomposites were evaluated [19]. The theoretical results showed a good agreement with the experimental data for polymer/SiO_2_ nanocomposites in that study.

In our previous study [20], it was found that the tensile strength of interphase layers changes by a power function of the distance between nanoparticles and polymer matrix. It was also shown that the calculations of this equation depend on *Z* parameter which shows the interphase properties. Additionally, it was discussed that the extent of *Z* determines the level of mechanical properties in the polymer nanocomposites. In this paper, the *Z* interphase parameter is defined by the material and interphase properties in polymer nanocomposites. The Pukanszky model and many useful equations are applied which only need to tensile strength of polymer nanocomposites and the properties of nanocomposite components. The dependency of *Z* to different material and interphase characteristics are illustrated by contour plots based on the resultant equations. The obtained results for different types of polymer nanocomposites are also explained by practical views in this area.

## Methods

**Fig. 1:**
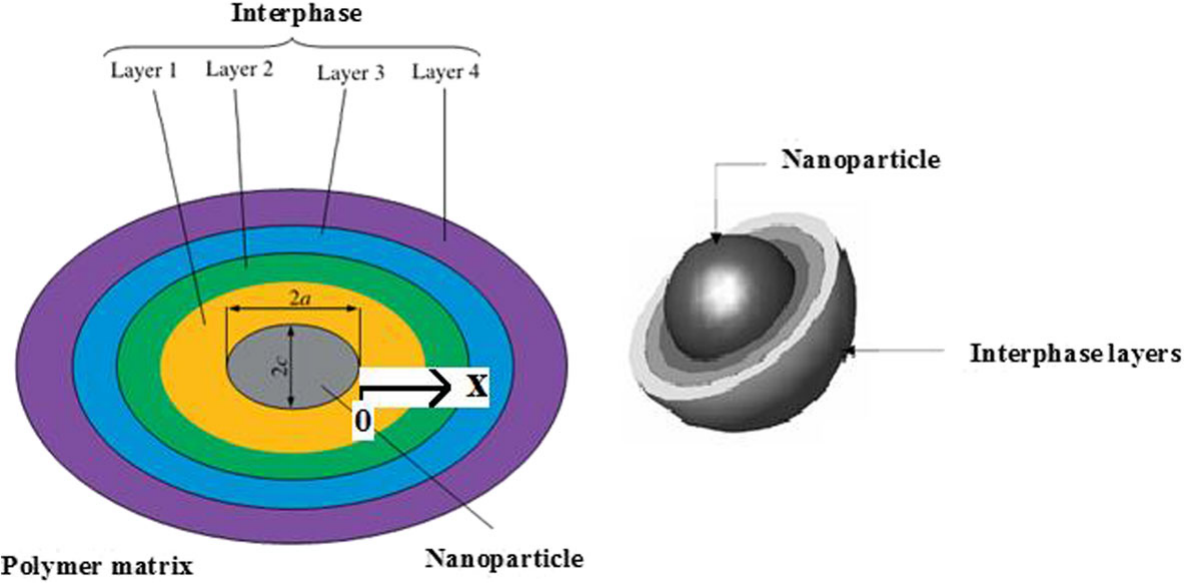
Graphic design of interphase layers around a nanoparticle in polymer nanocomposites

The general properties of the interphase between polymer matrix and nanoparticles such as coefficient of thermal expansion, stiffness, and strength depend on the properties of nanoparticles and polymer matrix. The interphase can be divided into *n* layers, where each layer has different properties (Fig. 1). Assuming a same thickness for interphase layers, the thickness of the *k* ^th^ layer is expressed by:

1 $$ {t}_k=\frac{t}{n} $$

where *t* is the total thickness of interphase. *x* changes from a nanoparticle surface (*x* = 0) to polymer matrix (*x* = *t*). The *x* for central point of the *k* ^th^ layer is given by:

2 $$ {x}_k=k{t}_k-\frac{t_k}{2} $$

In our previous work [20], it was reported that the tensile strength of interphase layers changes as a power function of *x*_*k*_ as:

3 $$ {\sigma}_{\mathrm{k}}={\sigma}_{\mathrm{p}}-\left({\sigma}_{\mathrm{p}}-{\sigma}_{\mathrm{m}}\right){\left(\frac{x_k}{t}\right)}^Z $$

where *σ*_m_ and *σ*_p_ are the tensile strength of matrix and nanoparticles, respectively, and *Z* is an interphase parameter which shows the properties of interphase. This equation is true for all polymer nanocomposites containing well-dispersed nanoparticles. The average strength of interphase (*σ*_i_) can be assumed as the tensile strength of the central layer, i.e., *σ*_k_ at *x*_*k*_ = *t*/2. Accordingly, *σ*_i_ is defined as:

4 $$ {\sigma}_{\mathrm{i}}={\sigma}_{\mathrm{p}}-\left({\sigma}_{\mathrm{p}}-{\sigma}_{\mathrm{m}}\right){(0.5)}^Z $$

From rearranging of the above equation, *Z* can be expressed as:

5 $$ Z=-1.44 \ln \left(\frac{\sigma_{\mathrm{p}}-{\sigma}_{\mathrm{i}}}{\sigma_{\mathrm{p}}-{\sigma}_{\mathrm{m}}}\right) $$

Pukanszky [21] suggested a model which relates the tensile strength of composites to *B* interfacial parameter. Pukanszky model is stated as:

6 $$ {\sigma}_R=\frac{1-{\varphi}_{\mathrm{f}}}{1+2.5{\varphi}_{\mathrm{f}}} \exp \left(B{\varphi}_{\mathrm{f}}\right) $$

where *σ*_R_ is the relative tensile strength as *σ*_c_/*σ*_m_ (*σ*_c_ is the tensile strength of composite) and *φ*_f_ is the volume fraction of nanofiller. This model has been successfully applied for many types of polymer nanocomposites [22, 23]. This model can be well applied to nanocomposites when $\ln \left({\sigma}_R\frac{1+2.5{\varphi}_{\mathrm{f}}}{1-{\varphi}_{\mathrm{f}}}\right)$ vs. *φ*_f_ results in a line with *B* slope.

*B* which shows the level of interfacial adhesion between matrix and filler is presented as:

7 $$ B=\left(1+{A}_c{\rho}_{\mathrm{f}}t\right) \ln \left(\frac{\sigma_{\mathrm{i}}}{\sigma_{\mathrm{m}}}\right) $$

where *A*_*c*_ and *ρ*_f_ are the specific surface area and density of filler, respectively. By rearranging of the latter equation, *σ*_i_ can be formulated as:

8 $$ {\sigma}_{\mathrm{i}}={\sigma}_{\mathrm{m}} \exp \left(\frac{B}{1+{A}_c{\rho}_{\mathrm{f}}t}\right) $$

Substituting Eq. 8 into Eq. 5 results in:

9 $$ Z=-1.44 \ln \left[\frac{\sigma_{\mathrm{p}}-{\sigma}_{\mathrm{m}} \exp \left(\frac{B}{1+{A}_c{\rho}_{\mathrm{f}}t}\right)}{\sigma_{\mathrm{p}}-{\sigma}_{\mathrm{m}}}\right] $$

which relates the *Z* parameter to material and interfacial/interphase characteristics.

Additionally, *A*_*c*_ can be defined for spherical (1), layered (2), and cylindrical (3) nanoparticles as:

10 $$ {A}_{c1}=\frac{A}{m}=\frac{A}{\rho_{\mathrm{f}}v}=\frac{4\pi {r}^2}{\rho_{\mathrm{f}}\frac{4}{3}\pi {r}^3}=\frac{3}{\rho_{\mathrm{f}}r} $$

11 $$ {A}_{c2}=\frac{A}{m}=\frac{A}{\rho_{\mathrm{f}}v}\cong \frac{2{l}^2}{\rho_{\mathrm{f}}{l}^2d}=\frac{2}{\rho_{\mathrm{f}}d} $$

12 $$ {A}_c=\frac{A}{m}=\frac{A}{\rho_{\mathrm{f}}v}=\frac{2\pi rl}{\rho_{\mathrm{f}}\pi {r}^2l}=\frac{2}{\rho_{\mathrm{f}}r} $$

where *A*, *m*, *v*, and *l* are the surface area, mass, volume, and length of particles, respectively. *r* and *d* are also the radius and thickness of nanofiller.

By substituting Eqs. 10, 11, and 12 into Eq. 9, *Z* is expressed for different nanocomposites as:

13 $$ {Z}_1=-1.44 \ln \left[\frac{\sigma_{\mathrm{p}}-{\sigma}_{\mathrm{m}} \exp \left(\frac{B}{1+3\frac{t}{r}}\right)}{\sigma_{\mathrm{p}}-{\sigma}_{\mathrm{m}}}\right] $$

14 $$ {Z}_2=-1.44 \ln \left[\frac{\sigma_{\mathrm{p}}-{\sigma}_{\mathrm{m}} \exp \left(\frac{B}{1+\frac{2t}{d}}\right)}{\sigma_{\mathrm{p}}-{\sigma}_{\mathrm{m}}}\right] $$

15 $$ {Z}_3=-1.44 \ln \left[\frac{\sigma_{\mathrm{p}}-{\sigma}_{\mathrm{m}} \exp \left(\frac{B}{1+2\frac{t}{r}}\right)}{\sigma_{\mathrm{p}}-{\sigma}_{\mathrm{m}}}\right] $$

Additionally, the volume fractions of interphase in different polymer nanocomposites are given by:

16 $$ {\varphi}_{\mathrm{i}1}={\varphi}_{\mathrm{f}}\left[{\left(\frac{r+t}{r}\right)}^3-1\right] $$

17 $$ {\varphi}_{\mathrm{i}2}={\varphi}_{\mathrm{f}}\left(\frac{2t}{d}\right) $$

18 $$ {\varphi}_{\mathrm{i}3}={\varphi}_{\mathrm{f}}\left[{\left(\frac{r+t}{r}\right)}^2-1\right] $$

As a result, *B* parameter is expressed by *φ*_i_ and *φ*_f_ for dissimilar samples as:

19 $$ {B}_1=\left[3{\left(\frac{\varphi_{\mathrm{i}}}{\varphi_{\mathrm{f}}}+1\right)}^{1/3}-2\right] \ln \left(\frac{\sigma_{\mathrm{i}}}{\sigma_{\mathrm{m}}}\right) $$

20 $$ {B}_2=\left(1+\frac{\varphi_{\mathrm{i}}}{\varphi_{\mathrm{f}}}\right) \ln \left(\frac{\sigma_{\mathrm{i}}}{\sigma_{\mathrm{m}}}\right) $$

21 $$ {B}_3=\left[2{\left(\frac{\varphi_{\mathrm{i}}}{\varphi_{\mathrm{f}}}+1\right)}^{1/2}-1\right] \ln \left(\frac{\sigma_{\mathrm{i}}}{\sigma_{\mathrm{m}}}\right) $$

By rearranging Eqs. 19, 20, and 21, *σ*_i_ can be expressed for different nanocomposites as:

22 $$ {\sigma}_{\mathrm{i}1}={\sigma}_{\mathrm{m}} \exp \left[\frac{B}{3{\left(\frac{\varphi_{\mathrm{i}}}{\varphi_{\mathrm{f}}}+1\right)}^{1/3}-2}\right] $$

23 $$ {\sigma}_{\mathrm{i}2}={\sigma}_{\mathrm{m}} \exp \left(\frac{B}{1+\frac{\varphi_{\mathrm{i}}}{\varphi_{\mathrm{f}}}}\right) $$

24 $$ {\sigma}_{\mathrm{i}3}={\sigma}_{\mathrm{m}} \exp \left[\frac{B}{2{\left(\frac{\varphi_{\mathrm{i}}}{\varphi_{\mathrm{f}}}+1\right)}^{1/2}-1}\right] $$

The *Z* parameter for different nanocomposites can be given by substituting Eqs. 22, 23, and 24 into Eq. 5 as:

25 $$ {Z}_1=-1.44 \ln \left[\frac{\sigma_{\mathrm{p}}-{\sigma}_{\mathrm{m}} \exp \left(\frac{B}{3{\left(\frac{\varphi_{\mathrm{i}}}{\varphi_{\mathrm{f}}}+1\right)}^{1/3}-2}\right)}{\sigma_{\mathrm{p}}-{\sigma}_{\mathrm{m}}}\right] $$

26 $$ {Z}_2=-1.44 \ln \left[\frac{\sigma_{\mathrm{p}}-{\sigma}_{\mathrm{m}} \exp \left(\frac{B}{1+\frac{\varphi_{\mathrm{i}}}{\varphi_{\mathrm{f}}}}\right)}{\sigma_{\mathrm{p}}-{\sigma}_{\mathrm{m}}}\right] $$

27 $$ {Z}_3=-1.44 \ln \left[\frac{\sigma_{\mathrm{p}}-{\sigma}_{\mathrm{m}} \exp \left(\frac{B}{2{\left(\frac{\varphi_{\mathrm{i}}}{\varphi_{\mathrm{f}}}+1\right)}^{1/2}-1}\right)}{\sigma_{\mathrm{p}}-{\sigma}_{\mathrm{m}}}\right] $$

which correlate the *Z* parameter to volume fractions of nanofiller and interphase in polymer nanocomposites.

## Results and Discussion

The effects of material and interphase properties on the *Z* parameter are explained according to the proposed equations by contour plots which illustrate the *Z* as functions of different variables.

**Fig. 2:**
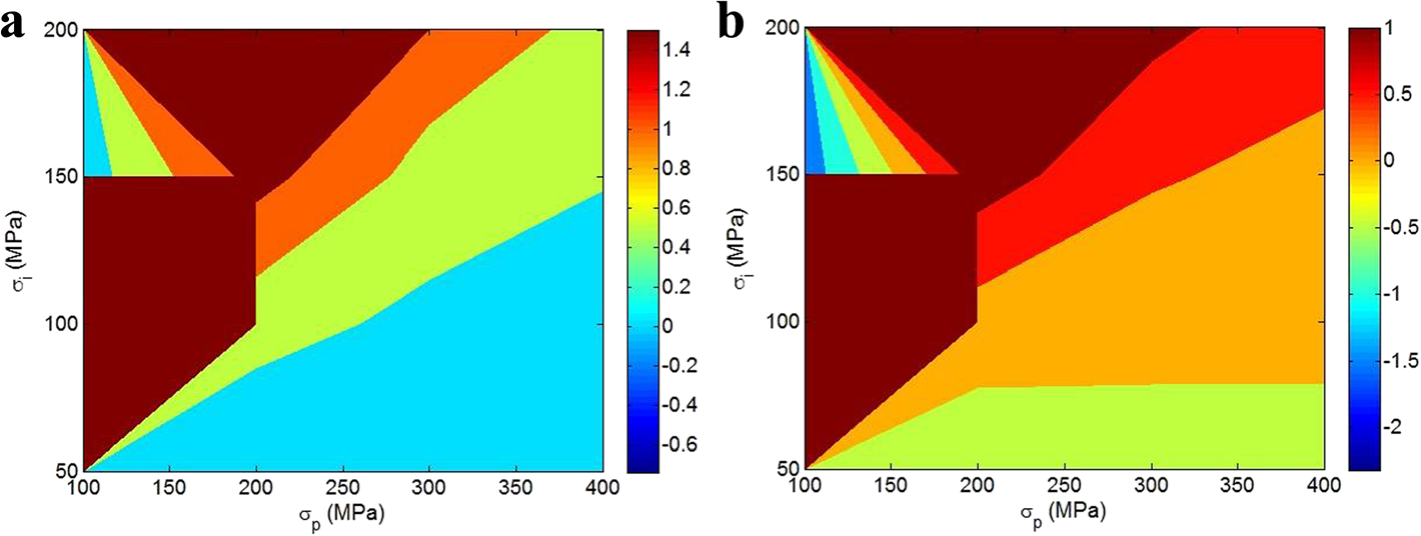
Contour plots to show the roles of *σ*_p_ and *σ*_i_ in *Z* parameter by Eq. 5 at **a** *σ*_m_ = 40 MPa and **b** *σ*_m_ = 80 MPa

Figure 2 illustrates the effects of *σ*_p_ and *σ*_i_ on the *Z* parameter (Eq. 5) at two various values of *σ*_m_. At *σ*_m_ = 40 MPa (Fig. 2a), a high level of *Z* is commonly obtained by a high *σ*_i_. It is understood that a strong interphase and a high *Z* show a direct relation which confirms the dependency of the *Z* to interphase properties. Also, it is obvious that a high *σ*_p_ suggests a small *Z*. As a result, the positive and negative roles of *σ*_i_ and *σ*_p_ in *Z* parameter are derived by this illustration.

The different effect of a very high *σ*_i_ and a very low *σ*_p_ on *Z* is not correct, because *σ*_i_ > *σ*_p_ cannot be practically occurred in polymer nanocomposites. The calculated *Z* at *σ*_m_ = 80 MPa (Fig. 2b) also show similar values to those of *σ*_m_ = 40 MPa. Accordingly, a high *Z* is obtained by high interphase strength (*σ*_i_) and low level of *σ*_p_ at different strength of matrix demonstrating the different influences of *σ*_i_ and *σ*_p_ on *Z* parameter.

**Fig. 3:**
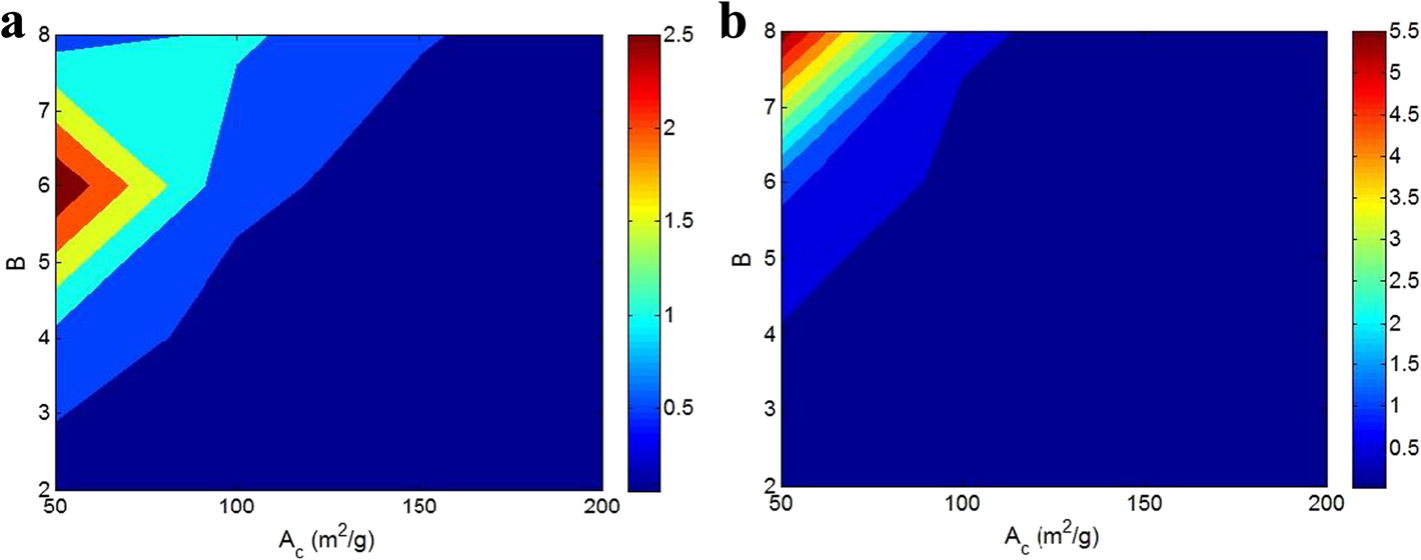
*Z* parameter as a function of *A*_*c*_ and *B* by Eq. 9 at *σ*_m_ = 40 MPa, *t* = 20 nm, *ρ*_f_ = 3 g/cm^3^ and **a** *σ*_p_ = 200 MPa and **b** *σ*_p_ = 300 MPa

Figure 3 shows the *Z* parameter as a function of *A*_*c*_ and *B* at two *σ*_p_ values based on Eq. 9 at *σ*_m_ = 40 MPa, *t* = 20 nm, and *ρ*_f_ = 3 g/cm^3^. At *σ*_p_ = 200 MPa (Fig. 3a), the highest values of *Z* are obtained by the lowest level of *A*_*c*_ at *B* = 6. The other values of *A*_*c*_ and *B* show a decreased *Z* parameter in this condition. The lowest values of *Z* are also reported by very low *B* (*B* < 3) or by very high *A*_*c*_ (*A*_*c*_ > 150 m^2^/g) at all extents of another parameter. As a result, *A*_*c*_ and *B* show dissimilar effects on the *Z* parameter and the other factors which define the values of *B* such as *t* and *σ*_i_ (Eq. 7) determine the final level of *Z*.

At *σ*_p_ = 300 MPa (Fig. 3b), it is found that both the highest *B* value and the lowest *A*_*c*_ level cause the best value of *Z*. Also, *B* < 4 or *A*_*c*_ > 120 m^2^/g suggests the smallest *Z* value at all levels of another parameter. In this condition, an increase in *A*_*c*_ and a decrease in *B* reduce the value of *Z*. It is obvious that the *A*_*c*_ plays a negative role in the value of *Z* at different *σ*_p_, but the high levels of *B* parameter show various *Z* attributed to the value of *σ*_p_. Therefore, the value of *σ*_p_ plays a critical role in the final level of *Z* at different *A*_*c*_ and *B* extents. The *Z* parameter shows a direct link with *B* parameter which expresses the level of interfacial adhesion in nanocomposite. Additionally, the high levels of *Z* and *B* significantly increase the level of *σ*_R_ in polymer nanocomposites (see Eqs. 4 and 6). So, the expression of *Z* parameter as an interphase parameter is true.

**Fig. 4:**
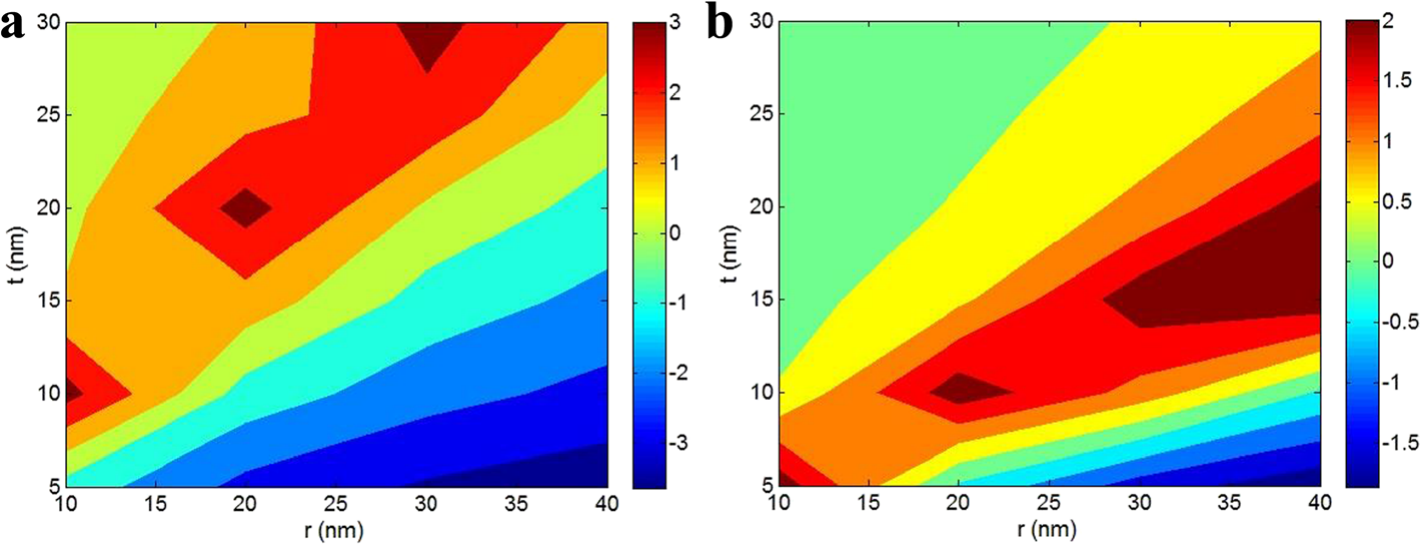
Contour plots for the influences of *r* and *t* on *Z* parameter in nanocomposites containing spherical nanoparticles (Eq. 13) at *B* = 5, *σ*_m_ = 40 MPa and **a** *σ*_p_ = 150 MPa and **b** *σ*_p_ = 360 MPa

The effects of *r* and *t* on the *Z* parameter in nanocomposites containing spherical nanoparticles (Eq. 13) are illustrated in Fig. 4 at *B* = 5, *σ*_m_ = 40 MPa, and different *σ*_p_. In the first state in Fig. 4a (*σ*_p_ = 150 MPa such as for SiO_2_), a negative *Z* is calculated by bigger nanoparticles and thin interphase (low *t*). The best levels of *Z* are obtained when both *r* and *t* have similar values. It means that the bigger nanoparticles show a great *Z* by a thick interphase, while the smallest nanoparticles can introduce a good *Z* by a thin interphase (less *t*). This evidence demonstrates that the smaller nanoparticles and the thicker interphase can suggest a high *Z* parameter in this condition. The smaller nanoparticles make a large interfacial area between polymer and nanoparticles, and a thick interphase provides a high level of *B* (see Eq. 7). Therefore, they can improve the mechanical properties of nanocomposites which may be expressed by a high *Z* parameter in this study.

The effects of *r* and *t* on *Z* parameter are also plotted in Fig. 4b when *σ*_p_ = 360 MPa such as for TiO_2_. In this condition, the negative effect of bigger nanoparticles on *Z* parameter is also illustrated similar to the previous situation, but the *t* plays different role compared to the former condition. In this state, the best levels of *Z* are achieved by *r* = 10 nm and *t* = 5 nm, *r* = 20 nm and *t* = 10 nm, and the bigger nanoparticles at medium level of *t* (14 < *t* < 21 nm).

These observations indicate that the size of nanoparticles and interphase differently affect the *Z* parameter based on the value of *σ*_p_. In other words, a high *σ*_p_ dictates different roles for nanoparticle size and interphase thickness in the level of *Z* parameter. Accordingly, an optimization should be performed in this case based on the type of nanofiller used in nanocomposite.

**Fig. 5:**
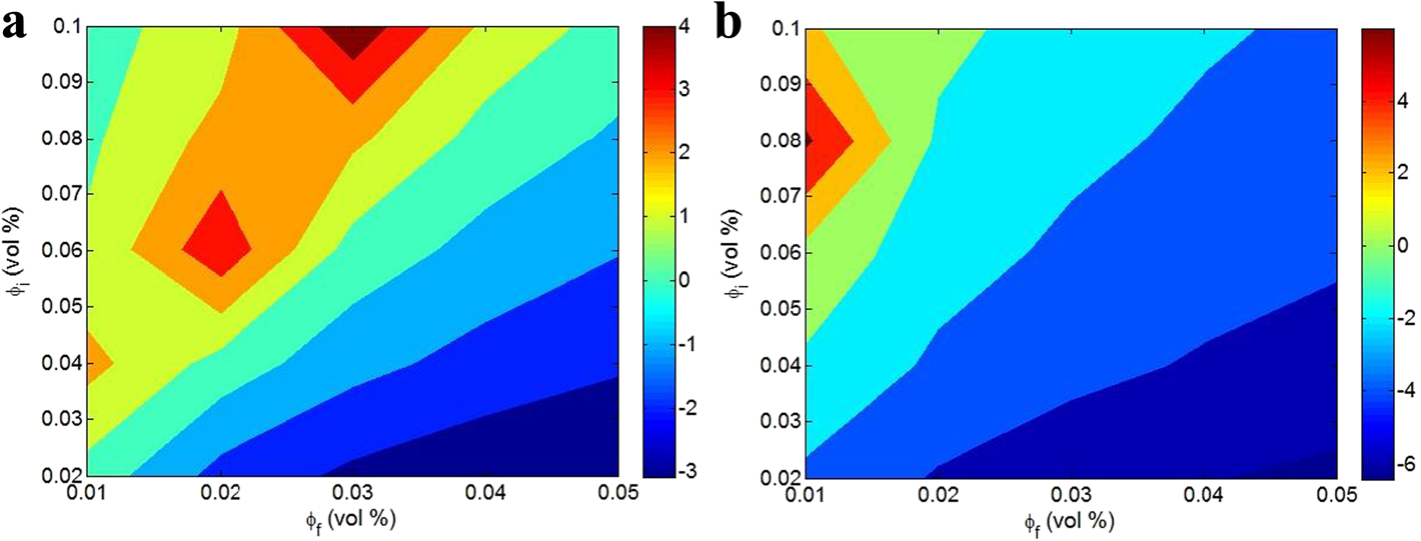
*Z* parameter as a function of *φ*_*i*_ and *φ*_*f*_ for nanocomposites reinforced with cylindrical nanofiller (Eq. 27) at *σ*_m_ = 40 MPa, *σ*_p_ = 200 MPa and **a** *B* = 5 and **b** *B* = 8

Figure 5 illustrates the roles of *φ*_i_ and *φ*_f_ in *Z* parameter for nanocomposites reinforced with cylindrical nanofiller (Eq. 27) at *σ*_m_ = 40 MPa, *σ*_p_ = 200 MPa, and different *B* values. When *B* = 5 (Fig. 5a), the high fractions of nanofiller as well as the low contents of interphase produce a negative *Z* value. Also, very high *φ*_i_ and very low *φ*_f_ which create a low *Z* are not practically obtained in polymer nanocomposites (see Eqs. 16, 17, and 18). In this condition (*B* = 5), the best values of *Z* are found at high *φ*_*i*_ and medium values of *φ*_f_. The predictions of this illustration are correct from practical points of view. A high *φ*_i_ positively contributes to the strengthening of polymer nanocomposite, due to the main role of the interphase properties in the final behavior of polymer nanocomposites [14, 24]. Also, a high *φ*_f_ typically introduces undesirable properties in nanocomposites, due to the aggregation of nanoparticles at high contents which decreases the interfacial area and promotes the stress concentration in polymer nanocomposites [25, 26].

At *B* = 8 (Fig. 5b), the same roles of *φ*_f_ and *φ*_i_ in *Z* value are also shown. However, a high *φ*_i_ at the lowest level of *φ*_f_ causes the best *Z*, while the former condition (*B* = 5) exhibits the best values of *Z* at high *φ*_i_ and medium values of *φ*_f_. This occurrence gives the different effects of *φ*_i_ and *φ*_f_ on *Z* parameter attributed to *B* parameter. Conclusively, the value of *B* plays a main role in the calculated results of *Z* by the suggested equations which should be considered in experiments.

## Conclusions

The *Z* interphase parameter for the tensile strength of interphase layers was expressed by material and interphase properties. The Pukanszky model for tensile strength of polymer nanocomposites was applied, and the dependency of *Z* to characteristics of constituents and interphase were explained by contour plots.

The main results reported in this article can be summarized as:

- The *σ*_i_ and *σ*_p_ show positive and negative roles in *Z* parameter at all values of *σ*_m_, respectively.
- The *σ*_i_ and *B* reveal direct links with *Z* parameter.
- The *A*_*c*_ plays a negative role in *Z* value at different *σ*_p_, but the dependency of *Z* to *B* parameter is associated with the value of *σ*_p_. Therefore, *σ*_p_ affects the final level of *Z* at different *A*_*c*_ and *B*.
- The *d* and *t* affect the *Z* parameter based on the value of *σ*_p_ in different manners. A high *σ*_p_ causes different roles for nanoparticle size and interphase thickness in *Z* parameter.
- The volume fractions of nanofiller and interphase dissimilarly affect the *Z* parameter. A high *Z* is obtained by a low nanoparticle volume fraction and a high content of interphase, but the best *Z* is obtained based on the level of *B* parameter.

## Abbreviations

*φ*_*f*_: Volume fraction of filler
*A*_*c*_: Specific surface area of filler
*B*: Interfacial adhesion parameter
*d*: Thickness of nanofiller
*k*^th^: Number of interphase layer
*r*: Filler radius
*t*: Total thickness of interphase
*x*: Distance
*Z*: Interphase parameter
*ρ*_f_: Density of filler
*σ*_c_: Tensile strength of composite
*σ*_i_: Strength of interphase
*σ*_m_: Tensile strength of matrix
*σ*_p_: Tensile strength of filler
*σ*_R_: Relative strength
